# Acute Thrombocytopenia Likely Induced by Apomorphine: A Case Report

**DOI:** 10.1002/mdc3.14253

**Published:** 2024-10-26

**Authors:** Clément Martinie De Maisonneuve, Valentin Mira, Romain Muller, Jean‐Philippe Azulay, Guillaume Hache

**Affiliations:** ^1^ Aix Marseille University, AP‐HM, Timone Hospital, Department of Pharmacy Marseille France; ^2^ AP‐HM, Timone Hospital, Department of Neurology and Pathology Of Movement Marseille France; ^3^ AP‐HM, Conception Hopital, Department of Clinic Immunology Marseille France

**Keywords:** neuropharmacology, Parkinson's disease, apomorphine, thrombocytopenia

Although drugs are a common cause of acute immune‐mediated thrombocytopenia in adults, they have rarely been observed with drugs used for the treatment of Parkinson's disease (PD), including in several cases on levodopa (l‐dopa). To the best of our knowledge, there is no report of thrombocytopenia induced by apomorphine in the literature.^1^, ^2^

## Case Report

A 79‐year‐old patient suffering from PD since 2004 presented motor fluctuations despite optimized oral treatment since 2023. She was hospitalized to initiate apomorphine pump in November 2023. Her treatment was l‐dopa/carbidopa/entacapone (100 mg/25 mg/200 mg, 5 times a day), l‐dopa/benserazide dispersible tablet (100 mg/25 mg upon awakening), ropinirol extended‐release tablet (16 mg per day), and amantadine (200 mg a day). During the month prior to hospitalization, 2 *off* episodes were treated with apomorphine pen injections (3 mg per injection). This treatment was discontinued because of nausea. She had no other condition except hypertension, treated by amlodipine. The month before the hospitalization, a blood test was performed and was found to have normal platelet count (278G/L) and a mild anemia (11.5 g/L).

Apomorphine pump flow rate was started at 1 mg/h over 10 h per day and then increased 0.5 mg/h up to a dose of 3.5 mg/h over 13 h per day (total daily apomorphine: 45.5 mg) on day 9. Domperidone (20 mg twice a day) was prescribed. We discontinued ropinirol and reduced l‐dopa/carbidopa/entacapone (to 75 mg/18.75 mg/200 mg, 4 times and 100 mg/25 mg/200 mg at bedtime). A blood sample was taken on the fifth day of hospitalization (apomorphine flow: 2.5 mg/h). It revealed moderate renal dysfunction (creatinine = 92 μmol/L [44–71 μmol/L], glomerular filtration rate = 66 mL/min/1.73 m^2^, and urea within normal limits) and isolated mild thrombocytopenia (105G/L). On day 9, she presented petechiae on her lower limbs, gingival bleeding, and an intrabuccal hemorrhagic bulla. No fever nor hemodynamic disturbance was noted. An emergency blood test found severe thrombocytopenia (2G/L) confirmed on citrated tube, associated with stable mild anemia without hemolysis (low reticulocyte count [49.8G/L], low lactate dehydrogenase, no schizocyte, normal haptoglobin). No evidence of platelet consumption was found (coagulation factor within normal range, d‐dimer not increased). Creatinine was stable.

Etiologically, we performed a blood test, including serologies, antinuclear antibodies, serum immunoelectrophoresis, thyroid assessment, folic acid, B12 vitamin, and glycoprotein‐specific antibody testing (anti‐GPIb/IIIa, anti‐GPIa/IIa, and anti‐GPIb/IX), which was normal. Bone marrow aspirations revealed rich marrow with abundant megakaryocytes and no abnormal cell. Given these data, and in the absence of heparin, we suspected apomorphine‐induced thrombocytopenia.

**FIG. 1:**
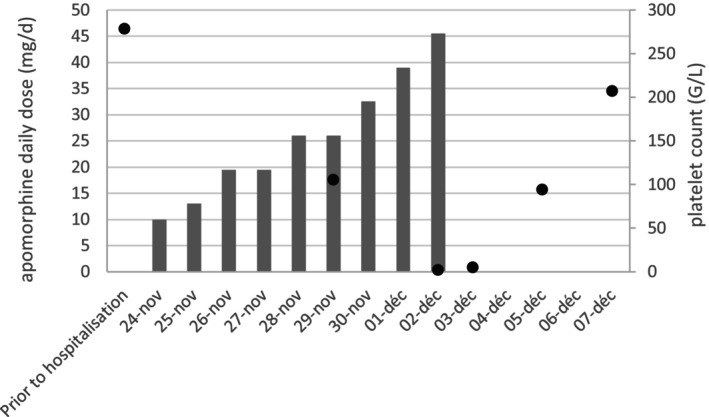
Time course evolution of both daily apomorphine dosage (histograms) and platelet count (line).

Therapeutically, we stopped apomorphine and administered corticosteroids 2 mg/kg/day for 4 days. No platelet transfusion was administered. We reintroduced the initial l‐dopa therapy. Evolution was favorable: petechiae disappeared, and the platelet count improved (207G/L) 5 days later, allowing patient discharge. Anemia persisted and was stable. We reduced corticosteroids to 1 mg/kg/day for 3 weeks. One month after corticosteroid withdrawal, she returned to her baseline. Biological tests showed a platelet count of 311G/L and persistent anemia (101 g/L). Clinicobiological data are summarized in Figure 1.

## Discussion

According to George and Aster's criteria, our patient is considered to be at the probable level of evidence of drug‐induced thrombocytopenia (DITP); drug administration preceded thrombocytopenia; recovery from thrombocytopenia was complete and sustained after drug discontinuation (criteria 1); other drugs administered prior to thrombocytopenia were continued after the discontinuation of apomorphine (criteria 2), and other etiologies were excluded (criteria 3).^3^ A comparable level of evidence was obtained according to Naranjo et al.'s algorithm, with a score of 6 corresponding to a probable level whose reaction was confirmed by the withdrawal of apomorphine.^4^

DITP is an idiosyncratic immune‐mediated reaction most often mediated by drug‐dependent antibodies resulting in increased platelet clearance. Various pathophysiological processes have been described to explain the appearance of such antibodies, including hapten‐induced antibody, “quinine‐type” antibody, drug‐specific antibody, fibrinogen receptor antagonist‐dependent antibody, autoantibody induction, and immune complexes.^5^ Compared to heparin‐induced thrombocytopenia where immune complexes are regularly found and easy to detect, leading to moderate thrombocytopenia in 5 to 10 days, other DITP typically has an abrupt onset of severe thrombocytopenia. Nadir platelet counts are often <20G/L, clinically important bleeding is common, and death from bleeding has been reported.^3^, ^5^ Typically, DITP occurs 1 to 2 weeks after beginning a new drug or suddenly after a single dose when a drug has previously been taken intermittently, which has been the case here.^3^ Recovery from thrombocytopenia begins within 1 or 2 days after drug discontinuation, and recovery is usually complete within a week. Here, we described a severe grade 3 adverse event according to CTCAEv5, and both clinical and biological recovery within 9 days after apormorphine discontinuation. Thus, we retained the diagnosis of DITP although we initially suspected a drug‐induced thrombopenic microangiopathy such as thrombotic thrombocytopenic purpura (TTP). However, thrombotic microangiopathy syndromes are defined by thrombocytopenia, microangiopathic hemolytic anemia, and microvascular thrombosis; we did not find any sign of hemolysis, and a normal ADAMTS13 activity excluded TTP.^6^

Apomorphine's hematological complications, notably hypereosinophilia and autoimmune hemolytic anemia, are uncommon.^7^ Whereas thrombocytopenia associated with l‐dopa has already been reported, thrombocytopenia associated with apomorphine infusion seems an extremely uncommon adverse effect.^8^ Interestingly, whereas thrombocytopenia associated with l‐dopa seemed to occur after long‐term use of l‐dopa, we described an abrupt thrombocytopenia a few days after apomorphine initiation.

## Author Roles

(1) Research project: A. Conception, B. Organization, C. Execution; (2) Statistical analysis: A. Design, B. Execution, C. Review and critique; (3) Manuscript: A. Writing of the first draft, B. Review and critique.

C.M.M.: 1C, 3A

V.M.: 1A, 1B, 3B

R.M.: 1B, 3B

J.‐P.A.: 3B

G.H.: 1A, 1B, 1C, 3B

## Disclosures

**Ethical Compliance Statement**: Given the anonymous nature of the data presented, institutional review board or ethics committee approval was not required for this case report. We have obtained written consent from the patient for this manuscript. We confirm that we have read the journal's position on issues involved in ethical publication and affirm that this work is consistent with those guidelines.

**Funding Sources and Conflicts of Interest**: No specific funding was received for this work. The authors declare that there are no conflicts of interest relevant to this work.

**Financial Disclosures for the Previous 12 Months**: The authors declare that there are no additional disclosures to report.

## Data Availability

The data that support the findings of this study are available from the corresponding author upon reasonable request.
